# Dental Students’ Knowledge and Attitudes about Human Papillomavirus Prevention

**DOI:** 10.3390/vaccines9080888

**Published:** 2021-08-11

**Authors:** Marjorie Wright, Vanessa Pazdernik, Corey Luebbering, Joan M. Davis

**Affiliations:** 1 College of Graduate Health Studies, AT Still University, Kirksville, MO 63501, USA; 2 Department of Research Support, AT Still University, Kirksville, MO 63501, USA; vpazdernik@atsu.edu; 3 Department of Research Support, AT Still University, Kirksville, MO 63501, USA; coreyluebbering@atsu.edu; 4 Missouri School of Dentistry and Oral Health, AT Still University, Kirksville, MO 63501, USA; joandavis@atsu.edu

**Keywords:** HPV, dental student, HPV vaccine

## Abstract

The purpose of the current study was to assess knowledge and attitudes about human papillomavirus (HPV) and HPV vaccination for oropharyngeal cancer (OPC) prevention in first-year through fourth-year dental students. Methods: In this cross-sectional quantitative study, surveys assessed knowledge and attitudes about HPV, HPV-related OPC, and HPV vaccination of incoming first-year dental students (D1), outgoing first- and second-year dental students (D1–D2), and outgoing third- and fourth-year dental students (D3–D4). After completing a 40-item pre-training knowledge and attitude assessment survey, a one-time, one-hour national HPV training session was conducted. An 8-item attitudinal post-survey was completed after training. Results: Of 173 participants (75.9% response rate), over 85% did not know that the rate of HPV is not highest among women in their 30s, and only 11% to 28% knew that smoking-associated OPC is more deadly than HPV-associated OPC. While participants overall expressed willingness to administer the HPV vaccine, the willingness of dental students to do so in their future practice tapered off progressively through dental school year categories (*p* < 0.001). Among outgoing D1–D4 students, the one-hour HPV training increased participants’ self-perceived ability to describe the burden of HPV disease, discuss the importance of HPV vaccination for cancer prevention, and provide needed HPV vaccination information to parents (all *p* ≤ 0.004). Conclusions: Understanding deficits in dental student knowledge and attitudes across the 4 years of dental school may help dental educators better understand the timing and content needed for effective HPV training in the dental school curriculum to reduce HPV-associated OPC prevalence.

## 1. Introduction

Human papillomavirus (HPV) is the most prevalent sexually transmitted disease in America [1] and the leading risk factor for oropharyngeal cancer (OPC) [2]. However, studies have found gaps in the knowledge and willingness of dental providers to discuss HPV and its vaccine with patients [3,4,5]. This may lead to missed opportunities for patient counseling and recommendation of HPV vaccination. Further, this outcome is concerning for dental educators seeking to instill future dental professionals with appropriate knowledge and attitudes about HPV.

The HPV vaccine has been effective at reducing the prevalence of HPV infection, including the high-risk HPV types 16 and 18 associated with oropharyngeal cancer (OPC) [6]. However, adherence to HPV vaccination guidelines from the Advisory Committee on Immunization Practices (ACIP) has been challenging. In 2016, ACIP guidelines revised the original three-dose vaccination recommendation to the current two doses for those aged less than 15 years [7,8]. Recommendations suggest that individuals aged 15–26 years receive three HPV vaccine doses and individuals aged 27–45 years should discuss this vaccination with their provider [8]. Of children aged 11–12 years, 68% received at least one dose of HPV vaccination in 2017–2018, but only 51% of adolescents had the entire two-dose or three-dose series [9].

The ACIP recommendations allow for HPV vaccination as early as 9 years old [8]. Further, children aged 9 years and older are more likely to visit the dentist than the physician [10], so dentists and other oral healthcare professionals have opportunities to discuss HPV and HPV vaccination with parents and patients. The American Dental Association [11], the American Academy of Pediatric Dentistry [12], and the American Association of Public Health Dentistry [13] recommend oral health providers provide education and counseling about HPV vaccination to prevent infection.

Despite the potential benefits of providing HPV vaccine counseling and recommendations in the dental office, studies of dentists [3,5,14,15], oral hygiene students [16,17], and dental hygienists [3,5,14,15] suggest gaps in HPV knowledge and reluctance to counsel or recommend HPV vaccination. One way to prevent these missed opportunities is to ensure dental school training prepares future professionals to be knowledgeable and comfortable with discussing and recommending HPV vaccination to patients. Therefore, the purpose of the current study was to assess and compare knowledge and attitudes about HPV and HPV vaccination for OPC prevention in first-year through fourth-year dental students. To our knowledge, our study is the first to assess knowledge and attitudes about HPV and HPV vaccination among dental students across all four dental school years, and our results may provide guidance for future development and integration of HPV counseling in dental school curricula.

## 2. Materials and Methods

### 2.1. Study Design and Participants 

The current study used a cross-sectional survey design, and all survey responses were anonymous. The study was conducted according to the guidelines of the Declaration of Helsinki, reviewed by the A.T. Still University Institutional Review Board and was deemed exempt. Electronic and paper-and-pencil surveys were completed by dental students before (pre-training) and after (post-training) a training session about HPV at two sites of a single US dental school. In March 2019, all outgoing first-year (D1), second-year (D2), third-year (D3), and fourth-year (D4) students were required to attend the one-time hour HPV training in collaboration with the Missouri Area Health Education Center HPV Immunization Project (a US Centers for Disease Control and Prevention-funded initiative). Each student was e-mailed a survey invitation that described the study and included a link to the electronic pre-training survey. The survey was compiled by the author JD and administered by the author CL. The first question of the 40-question survey addressed informed consent, and all students could choose to participate in the study or opt out. Students were advised that their participation was voluntary and that there were no negative consequences of non-participation. The one-hour training session provided instruction about HPV and the HPV vaccination. An onsite lunch was provided to encourage participation in the voluntary pre-training survey. After the one-hour training, dental students completed a voluntary 8-question post-training paper-and-pencil survey. In July 2019, incoming D1 students were invited to participate in the study by completing the electronic pre-training survey only. No training was provided at that time.

### 2.2. Survey Instrument 

The 40-question pre-training survey had three sections: demographics, 30 items assessing knowledge of HPV and OPC, and 10 items assessing attitudes about the scope and role of dental professionals in addressing HPV and OPC through counseling and administration of the HPV vaccine. Demographic items asked about sex, age, year in dental school, and race and ethnicity. Knowledge assessment included 18 items about HPV and OPC and 12 about HPV vaccination; response choices were (yes, no, or unsure) and one 5-level multiple choice. Attitude assessment asked about perceived responsibility, willingness, and understanding of HPV and OPC; these items used 4-point to 7-point Likert-like scales. An open text box was included to allow participants to provide comments or reflections about providing HPV vaccination information during a dental appointment. The students were given 30 min to complete the survey.

The 7-item post-training survey used 5-point Likert scale items that estimated the effect of the training session directly, i.e., “as a result of this training”, and further, some items also included modifiers such as “better” and “more aware” (1 = strongly agree and 5 = strongly disagree). An additional question requested feedback or comments. The students had 30 min to complete the survey following the HPV training. Double-data entry was used to convert paper responses to digital format for analysis. The two copies were also compared to resolve any inconsistencies.

Knowledge questions related to HPV and HPV-related oropharyngeal cancer and five self-perception HPV immunization administration questions were based on a validated questionnaire from Rutkoski et al. [18]. Five additional validated questions assessing self-perceived ability to provide HPV immunization counseling were used with permission from the Missouri Area Health Education Center HPV Immunization Project. Survey content was created and managed using Qualtrics (Qualtrics, Provo, UT, USA) survey software.

### 2.3. Data Analysis

Frequencies and percentages were calculated for survey responses. Fisher exact tests were used to assess differences between groups (incoming D1, outgoing D1–D2, and outgoing D3–D4) in proportions of a correct response for each of the 30 quiz questions. Blank responses found among thirteen participants and “not sure” responses were marked incorrect. Kruskal–Wallis and Mann–Whitney post hoc tests were used to assess differences between groups in attitude questions related to perceived responsibility, willingness, and understanding of HPV and OPC. For three post-training survey items that assessed improvement relative to pre-training directly, we used Wilcoxon signed rank tests to assess change among those who did not have the ability before the training session. For two post-training survey items assessing ability that was also assessed at pre-training, we used the Fisher exact test to compare outgoing D1–D4 pre- and post-participants as two independent groups. For purposes of comparison to pre-training assessment in these Fisher exact tests, the post-training responses that indicated their ability existed prior to training were considered as “strongly agree”. SAS version 9.4 software (SAS Institute, Inc., Cary, NC, USA) was used to conduct analyses. A *p* < 0.05 was considered statistically significant.

### 2.4. HPV Training Program

The Missouri Area Health Education Center HPV Immunization Project requested that the dental school provide an HPV education program for our dental students. Permission was given to JD to use the existing medically focused training program as a basis to develop a dental-specific one-hour training program. The training program included a PowerPoint presentation, informational handouts, and web addresses as resource materials. The PowerPoint presentation and handouts were sent to participants one day prior to the actual training.

## 3. Results

All 228 students enrolled at the dental school were sent pre-training survey invitations, 173 (75.9% response rate) completed *N*= 171) or partially completed (*N* = 2) the survey. Of these, 111 (64.2%) were completed by outgoing students and 62 (35.8%) by incoming D1 students. The demographic characteristics of study participants are presented in Table 1. 

Student responses to the pre-training survey are presented in Table 2.

In 14/30 questions, we found a significant difference between groups in the proportions of correct responses (all *p* < 0.048). In all these questions, fewer incoming D1 students answered correctly compared with outgoing D1–D2 and/or outgoing D3–D4 (all post hoc *p* < 0.046). About half of outgoing students and 29% of incoming D1 students correctly indicated the falsehood that “some types of HPV can cause HIV/AIDS”. Among outgoing students and incoming D1 students 25% and 11%, respectively, understood that OPC caused by smoking is more deadly than OPC caused by HPV. About 50% of outgoing students and 36% of incoming D1 students understood that HPV vaccines do not cause serious side effects. Between 25% of incoming D1 and 50% of D3–D4 knew 11–12 years is the recommended age for HPV vaccination in youth. Among outgoing students, in one question, more D1–D2 students knew that almost all cervical cancers are caused by HPV infection compared with D3–D4 students (73.6% (39/53) vs. 51.6% (30/58); post hoc *p* = 0.02). More consistent across groups, overall, only 14.5% (25/173) knew that the rate of HPV is not highest among women in their 30s and only 46.8% (81/173) knew that HPV vaccines do not have serious side effects. Knowledge of the association between HPV and OPC was well known with 86.1% (149/173) of students correctly indicating the link, and slightly less than half understood that people who have been diagnosed with HPV should not receive the HPV vaccine.

Nearly all students (98.2%; 167/170) at least somewhat agreed that both discussing and recommending HPV vaccination falls within the scope of a dental professional (Table 3).

The outgoing D3–D4 students tended to agree least with these two questions, and incoming D1 students had greatest agreement (both post hoc *p* < 0.006). Half (86/171) of all students indicated it was extremely important as a dentist to provide counseling to parents about HPV and its link to OPC. Incoming and outgoing D1–D2 students tended to rate the importance of HPV vaccination counseling more highly than D3–D4 students (both post hoc *p* < 0.002). Slightly less than half of all students indicated they were very willing to administer HPV (45.6%; 78/171) or flu (47.4%; 81/171) vaccines. Incoming D1 students tended to be more willing to vaccinate patients for HPV or flu than D3–D4 students (both post hoc *p* < 0.001). The most common response was “agree” to items on perceived understanding of HPV and OPC. Incoming D1 and outgoing D3–D4 students tended to agree less with possessing the ability to describe the burden of HPV disease than outgoing D1–D2 students (both post hoc *p* < 0.01). Similarly, incoming D1 students tended to agree less with possessing the ability to explain the rationale for vaccinating youth than outgoing D1–D2 students (post hoc *p* = 0.02). No differences were found for year in school for other questions about perceived understanding related to HPV and OPC (all *p* > 0.08).

Of 165 outgoing D1–D4 students, 143 (86.7% response rate) completed the post-training survey self-assessing their ability to provide HPV-related education and counseling as a result of the training session. Responses are summarized in Table 4. Following the one-hour HPV training, most participants agreed that they were better able to describe the burden of HPV disease (87% (125/143)), better able to explain the need to vaccinate youth ages 11 or 12 years (83% (119/143)), and more aware of CDC recommendations for administering the HPV vaccine (84% (120/143)) (all *p* < 0.001). After the training, including those who indicated they already possessed the ability, more agreement was found at post-training compared with pre-training for being able to define the importance of HPV vaccination for cancer prevention (post: 92% (131/143), pre outgoing: 83% (91/109); *p* = 0.004) and provide needed HPV vaccination information to parents (post: 91% (130/143), pre outgoing: 49% (53/109); *p* < 0.001).

## 4. Discussion

The purpose of the current study was to assess knowledge and attitudes about HPV and HPV vaccination for OPC prevention in first-year through fourth-year dental students. There were 14 questions where incoming D1 students were less knowledgeable than at least one of the outgoing groups. In the remaining 16 questions, there was not a statistically significant difference among the three groups. The willingness to administer the HPV vaccine successively decreased from incoming D1, to outgoing D1–D2, to outgoing D3–D4 students, who showed the least willingness to administer the vaccine. To our knowledge, our study was the first to investigate knowledge, attitudes, and intentions of dental students regarding HPV infection and vaccination across all 4 years of dental education.

### 4.1. Knowledge of HPV, HPV-Related OPC, and HPV Vaccine

Knowledge about HPV’s link to OPC was known among most students in the pre-test survey and did not show a statistically significant association with dental school year. Over 85% of students could not confirm women in their 30s are not the population with the highest rate of HPV, and this also did not show a statistically significant association with dental school year. Student responses also indicated a misunderstanding of HPV infection sequelae: only a minority knew that HIV/AIDS cannot result from an HPV infection. These findings should concern oral healthcare professionals. Providing accurate information about potential consequences of HPV infection can improve patient confidence in dental providers and position dental professionals as trusted experts on HPV. Therefore, dental professionals need to be educated and prepared to counsel patients and parents about HPV, its link to OPC, and administration of the HPV vaccine as a primary prevention strategy.

In terms of HPV vaccine knowledge, participants were less knowledgeable about serious side effects of the vaccine, the recommended age for vaccination, and the safety of vaccinating individuals with a past HPV diagnosis. These results should concern dental educators because they suggest that some students enter the dental workforce ill-prepared to provide accurate information to patients and parents about the HPV vaccination.

Overall, students in the current study were unaware that OPC caused by smoking is more deadly than HPV-associated OPC. Patel et al. [5] found similar results in their survey of dentists and dental hygienists, as did Rutkowski et al. [16] when surveying D3, D4, and dental hygiene students across multiple institutions. While it is important not to minimize the risk of HPV-related OPC, developers of dental school and continuing education curricula should include all risk factors of OPC when creating education materials.

### 4.2. Attitudes about HPV Vaccine

The majority of dental students in our study at least agreed that recommending the HPV vaccine to patients is within the scope of practice of dental professionals. Research suggests that parents of children with at least one dose of HPV vaccine had HPV vaccination recommended by a healthcare provider more often than parents of children who were not vaccinated [19]. In our study, outgoing D3–D4 students were least likely to strongly agree that HPV vaccination recommendations were within the scope of practice of dental providers, which suggested a possible lack of translation from D1–D2 didactic HPV education to clinical D3–D4 practice. If dental students are taught to feel comfortable making HPV vaccine recommendations to parents and guardians, we may be able to increase the number of vaccinated youth.

The majority of study participants strongly agreed that discussing the link between HPV and OPC was within the scope of practice of dental professionals. On the pre-training survey, less than half agreed or strongly agreed that they could adequately discuss the burden of HPV disease with patients or provide compelling enough information about the HPV vaccine to convince parents to vaccinate their children. Despite these results, a majority of students agreed that they were aware of the Centers for Disease Control and Prevention recommendations for HPV vaccination, the rationale for vaccinating youth aged 11–12 years, and the importance of HPV vaccination for cancer prevention.

The current study did not investigate why students felt unprepared to convince parents of the value of HPV vaccinations. Torres et al. [20] reported that only 11% of surveyed dental schools included patient education in their curricula. In a study by Cotter et al. [21], an educational module about HPV patient education and counseling increased confidence in the ability of oral health students to counsel patients about the HPV vaccine and to recommend vaccination. Similarly, after our HPV training session, most students agreed to each of the improvements or skills resulting from the training, including convincing parents of the value of HPV vaccination. As such, improved comfort and confidence of students when approaching parents about HPV could be addressed by re-evaluating the timing and content of HPV training in dental school by increasing exposure to and reinforcement of HPV vaccine benefits. The definitive increase in the percentage of dental students reporting ability to provide HPV-related counseling and health education to parents following one hour of HPV training holds promise. Future research should investigate student beliefs about their inability to convince parents to vaccinate children for HPV and clinical faculty comfort level when discussing HPV vaccinations with patients to ensure appropriate scenario modeling for students. Studies should also investigate the most effective oral health professional communication methods for successful vaccination, which may be useful for guiding the HPV-related dental school curriculum [19].

### 4.3. Willingness to Administer the HPV Vaccine

Most students in the pre-training survey were willing to administer the HPV vaccine in the dental office if properly trained. This finding is important for future dental practice. Although participants indicated for the most part that they were at least willing to train to administer the vaccine, Patel et al. [5] found dentists were generally neutral on administering it. In contrast, Harris et al. [22] found dentists were reluctant to administer the vaccine. Providing hands-on HPV vaccine administration training in dental schools might increase the comfort and willingness of those future dental professionals to vaccinate patients in the dental office.

Surprisingly, we found a progressive decrease in willingness or strong willingness of dental students to administer HPV vaccines in the dental office as students progressed through dental school. Our study did not investigate the reasons for dental student unwillingness to administer the HPV vaccine; however, Kepka et al. [17] reported role conflict as the reason most cited by dental students for their unwillingness to administer the HPV vaccine. Given the progressive reluctance to administer the HPV vaccine in our study, this result may reflect a change from idealism when starting dental school to realism when nearing graduation because of concerns about liability, accountability, and reimbursement. Administering HPV vaccines in the dental office can also require additional responsibilities of vaccine storage, inventory, and staff training, which are not necessarily addressed in dental school curricula.

Future research could investigate the effectiveness of HPV training longitudinally throughout the four-year dental curriculum on student knowledge and intent to administer and manage HPV vaccinations and storage, tailoring the curriculum to potentially alleviate reluctance of students to administer the vaccines as they get nearer to graduation. Studies could also investigate how educators can maintain student focus on promoting HPV vaccinations to parents and patients. The recent Executive Order allowing dentists to administer the COVID-19 vaccine signals policymaker acknowledgment that dentists can provide vaccinations. We need to ensure that our dental students are leaving dental school willing to do so.

### 4.4. Limitations

The current study had several limitations. The students were from a single dental school, so results are not generalizable to other populations or institutions. Another limitation is that incoming D1 students completed only the pre-training survey. Future studies should consider training and surveying these students regarding intent-to-vaccinate for comparisons with current dental students. Further, asking students about their intentions on the pre-training survey and then linking their pre-training and post-training survey responses may more definitively indicate the effectiveness of the HPV training session.

## 5. Conclusions

Participants nearer to graduation than those in earlier dental school years indicated they would be less willing to administer the HPV vaccine, despite showing sometimes more or no significant difference in nearly all knowledge scores about HPV or the HPV vaccine. Given the results of the current study, dental educators should evaluate their curricula for the timing and content of HPV training to ensure that graduating students are trained and willing to provide HPV vaccinations to reduce HPV infections and HPV-associated OPC. Future research should investigate the timing and content of different HPV-related curriculum models in dental schools for post-graduation willingness to administer HPV vaccines.

## Figures and Tables

**Table 1:** Demographic Characteristics (*N* = 173).

Demographic Characteristic	Pre-Survey, *N* (%) or Mean (SD)
Age ^a^	25.7 (3.4)
Gender	
Male	84 (48.6)
Female	89 (51.5)
Year in Dental School	
Incoming D1	62 (35.8)
Outgoing D1	27 (15.6)
Outgoing D2	26 (15.0)
Outgoing D3	31 (17.9)
Outgoing D4	27 (15.6)
Race and Ethnicity	
American Indian or Alaska Native	1 (0.6)
Asian	32 (18.5)
Latin American	6 (3.5)
White	123 (71.1)
Other	6 (3.5)
Prefer not to answer	5 (2.9)

^a^*N* = 172 because 1 student did not indicate.

**Table 2:** 30-Item knowledge assessment of HPV and HPV-OPC and HPV Vaccine (electronic pre-survey).

	*N*	Correct, *n* (%)					
		All	Incoming	Outgoing			
Question			D1 (*N* = 62)	D1 (*N* = 27)	D2 (*N* = 26)	D3 (*N* = 31)	D4 (*N* = 27)
**Questions on HPV and HPV-OPC**							
There are many types of HPV (human papillomavirus). * (Yes)	172	166 (97)	59 (97)	26 (96)	24 (92)	31 (100)	26 (96)
HPV is a bacterial infection. * (No)	172	157 (91)	51 (84)	24 (89)	26 (100)	30 (97)	26 (96)
A person can be infected with HPV without knowing it. *(Yes)	173	173 (100)	62 (100)	27 (100)	26 (100)	31 (100)	27 (100)
In most cases, HPV infections of all types go away before they cause any health problems. * (Yes)	172	45 (26)	5 (8)	10 (37)	6 (23)	13 (42)	11 (41)
HPV can be transmitted via sexual contact. * (Yes)	173	172 (99)	61 (98)	27 (100)	26 (100)	31 (100)	27 (100)
A person can transmit HPV even though a genital wart or lesion may not be present. * (Yes)	173	165 (95)	55 (89)	26 (96)	26 (100)	31 (100)	27 (100)
Some types of HPV are associated with approximately 70% of oropharyngeal cancers. * (Yes)	173	149 (86)	53 (85)	24 (89)	20 (77)	27 (87)	25 (93)
Most types of HPV are not harmful to people. * (Yes)	173	77 (45)	12 (19)	16 (59)	10 (38)	21 (68)	18 (67)
Some types of HPV cause HIV/AIDS. * (No)	172	74 (43)	18 (29)	12 (44)	13 (52)	14 (45)	17 (63)
The same types of HPV that infect the genital areas can infect the mouth and throat. * (Yes)	173	153 (88)	56 (90)	24 (89)	22 (85)	26 (84)	25 (93)
Genital warts are caused by the same HPV types that cause cervical cancer. * (No)	173	50 (29)	11 (18)	10 (37)	10 (38)	9 (29)	10 (37)
Almost all cervical cancers are caused by HPV infection. * (Yes)	172	101 (59)	32 (52)	19 (73)	20 (77)	14 (45)	16 (59)
The rate of HPV is highest among women in their 30s. * (No)	173	25 (14)	11 (18)	2 (7)	4 (15)	6 (19)	2 (7)
HPV related dysplasias occur more commonly in smokers. * (Yes)	172	98 (57)	26 (42)	20 (74)	16 (62)	16 (53)	20 (74)
Using a condom or dental dam will decrease the chance of transmitting HPV. * (Yes)	173	146 (84)	48 (77)	22 (81)	24 (92)	28 (90)	24 (89)
Antibiotics can cure HPV. * (No)	172	147 (85)	45 (74)	24 (89)	25 (96)	27 (87)	26 (96)
Oropharyngeal cancer caused by smoking is more deadly than oropharyngeal cancer caused by HPV. * (Yes)	172	37 (22)	7 (11)	10 (37)	5 (19)	5 (16)	10 (37)
Early stages of HPV oropharyngeal cancer are often asymptomatic. * (Yes)	172	146 (85)	41 (67)	27 (100)	25 (96)	26 (84)	27 (100)
**Questions on HPV Vaccine**							
There are vaccines that provide immunity against certain types of HPV. * (Yes)	170	163 (96)	58 (94)	26 (100)	24 (96)	29 (97)	26 (96)
HPV vaccines lead to long lasting immunity. * (Yes)	171	126 (74)	38 (61)	22 (81)	23 (92)	24 (80)	19 (70)
HPV vaccines can protect women against cervical cancer. * (Yes)	171	150 (88)	48 (77)	25 (93)	24 (96)	29 (97)	24 (89)
HPV vaccines protect men and women against oropharyngeal cancer. * (Yes)	171	123 (72)	40 (65)	21 (78)	19 (76)	24 (80)	19 (70)
Existing HPV vaccines do not protect an individual from all types of HPV. * (Yes)	171	143 (84)	48 (77)	23 (85)	20 (80)	27 (90)	25 (93)
HPV vaccines may cause serious side effects. * (No)	171	81 (47)	22 (35)	16 (59)	14 (56)	16 (53)	13 (48)
HPV vaccines are covered by most insurance providers. * (Yes)	171	122 (71)	41 (66)	23 (85)	19 (76)	20 (67)	19 (70)
HPV vaccines are administered in one dose. * (No)	171	92 (54)	28 (45)	14 (52)	16 (64)	13 (43)	21 (78)
The recommended age for HPV vaccination in youth is subjects aged: *(11–12 years)	169	65 (38)	15 (25)	10 (37)	12 (48)	14 (47)	14 (52)
HPV vaccines prevent 90% of individuals from getting genital warts. * (Yes)	169	99 (59)	32 (53)	18 (67)	14 (56)	19 (63)	16 (59)
People who have been diagnosed with HPV should not receive the HPV vaccines. * (No)	171	77 (45)	27 (44)	14 (52)	9 (36)	16 (53)	11 (41)
The Centers for Disease Control and Prevention (CDC) recommends that the HPV vaccines should be administered to both males and females. * (Yes)	170	155 (91)	56 (92)	25 (93)	22 (88)	27 (90)	25 (93)

Abbreviations: OPC, oropharyngeal/head and neck cancer. * correct answer not shown in the survey.

**Table 3:** Perceived Responsibility, Willingness, and Understanding of HPV and OPC (electronic pre-survey).

Scheme	Questions	*N* (%)							*p*
Pre (*N* = 171)		Strongly Agree	Agree	Somewhat Agree	Neither Agree nor Disagree	Somewhat Disagree	Disagree	Strongly Disagree	
	Discussing the link between HPV and oropharyngeal cancer falls within the scope and role of a dental professional. ^a^	112 (66)	46 (27)	9 (5)	3 (2)	0 (0)	0 (0)	0 (0)	<0.001
	Recommending HPV vaccination falls within the scope and role of a dental professional.	84 (49)	55 (32)	18 (11)	10 (6)	3 (2)	1 (1)	0 (0)	0.007
		Extremely important	Very important	Moderately important	Slightly important	Not at all important			
	How important is it for you as a dentist to provide counseling to parents about HPV vaccine and HPV’s link to oral cancer?	86 (50)	49 (29)	25 (15)	11 (6)	0 (0)			<0.001
		Very willing	Willing	Not willing	Other				
	If properly trained, how willing would you be to administer the HPV vaccines in your dental office? ^a^	78 (46)	58 (34)	32 (19)	2 (1)				<0.001
	If properly trained, how willing would you be to administer the flu vaccine in your dental office? ^a^	81 (48)	54 (32)	34 (20)	1 (1)				<0.001
		Strongly agree	Agree	Neither agree nor disagree	Disagree	Strongly disagree			
	I am currently able to describe the burden of HPV disease.	20 (12)	60 (35)	56 (33)	32 (19)	3 (2)			0.01
	I am currently able to provide useful and compelling information about the HPV vaccine to parents to aid in making the decision to vaccinate. ^a^	22 (13)	61 (36)	48 (28)	36 (21)	3 (2)			0.27
	I am aware of the CDC recommendations for administering the HPV vaccine to both boys and girls.	58 (34)	68 (40)	22 (13)	22 (13)	1 (1)			0.29
	I am currently able to explain the rationale for vaccinating youth at ages 11 or 12. ^a^	32 (19)	67 (39)	40 (24)	26 (15)	5 (3)			0.052
	I can define the importance of HPV vaccination for cancer prevention.	46 (27)	87 (51)	22 (13)	14 (8)	2 (1)			0.18

^a^*N* = 170 because 1 student did not indicate. Differences among dental students using Kruskal–Wallis test.

**Table 4:** Responses to post-survey.

Question	*N*	Strongly Agree	Agree	Neither Agree nor Disagree	Disagree	Strongly Disagree	NA
As a result of this training, I am better able to describe the burden of HPV disease.	143	52 (36)	73 (51)	10 (7)	1 (1)	1 (1)	6 (4)
As a result of this training, I can define the importance of HPV vaccination for cancer prevention.	143	55 (38)	70 (49)	6 (4)	2 (1)	0 (0)	10 (7)
As a result of this training, I am better able to explain the rationale for vaccinating youth at ages 11 or 12.	143	49 (34)	70 (49)	9 (6)	1 (1)	0 (0)	14 (10)
As a result of this training, I am more aware of the CDC recommendations for administering the HPV vaccine to both boys and girls.	143	60 (42)	60 (42)	8 (6)	2 (1)	0 (0)	13 (9)
As a result of this training, I can provide useful and compelling information about the HPV vaccine to parents to aid in making the decision to vaccinate.	143	53 (37)	70 (49)	11 (8)	2 (1)	0 (0)	7 (5)
As a result of this training, I can locate resources relevant to current immunization practice.	143	43 (30)	83 (58)	8 (6)	3 (2)	1 (1)	5 (3)
For Students Only: The content and instructional materials presented met my educational needs regarding HPV vaccinations.	140	54 (39)	73 (52)	10 (7)	2 (1)	0 (0)	1 (1)

Abbreviations: NA, not applicable; NA indicates that the post-survey question was not relevant, or ability existed prior to training.

## Data Availability

Please contact the corresponding author for supporting data.
